# Beyond metabolism and blood flow: toward an inflammation-metabolism axis paradigm for herbal medicine in obesity-related coronary heart disease

**DOI:** 10.3389/fcvm.2026.1806765

**Published:** 2026-06-18

**Authors:** Jia-mei Fu, Xi-ze Sun, Zi-yi Guo, Qi-yue Zhao, Ya-xiong Sun, Hai-xiang Li, Xiao-hong Yu

**Affiliations:** 1 Second Department of Cardiology, First Afliated Hospital of Heilongjiang University of Chinese Medicine, Harbin, China; 2 Postdoctoral Research Station of Heilongjiang University of Chinese Medicine, Harbin, China; 3 Health Preservation and Healthcare in Chinese Medicine, Baicheng Medical College, Baicheng, China; 4 The First School of Clinical Medicine, Heilongjiang University of Chinese Medicine, Harbin, China; 5 Department of Cardiology, Hohhot Hospital of Traditional Chinese Medicine and Mongolian Medicine, Hohhot, China; 6 Department of Chinese Medicine, Danzhou People's Hospital, Danzhou, China

**Keywords:** coronary artery disease, herbal medicine, immunometabolism, metabolic inflammation, multi-target therapy, network pharmacology, obesity, residual cardiovascular risk

## Abstract

Patients with obesity-related coronary heart disease continue to experience substantial residual cardiovascular risk despite progress in secondary prevention. We propose that this risk originates from a self-perpetuating cycle between inflammation and metabolism—termed the inflammation-metabolism axis—which extends beyond conventional risk factors. This axis forms a complex pathological network that existing single-target therapies cannot adequately address, representing a core mechanism of persistent residual risk. Effective management therefore requires integrated strategies capable of systemically modulating this network. Herbal medicines, with their inherent multi-target properties and network-level activity, offer a dual opportunity: they act as both potential therapeutic agents and as scientific tools for validating this integrative paradigm. This perspective article outlines the inflammation-metabolism axis as a unifying framework and discusses the rationale and future directions for developing systemic herbal-based interventions to mitigate residual cardiovascular risk.

## 1 Introduction

Despite considerable progress in the secondary prevention of atherosclerotic cardiovascular disease, particularly through intensive lipid-lowering and antiplatelet therapies, patients with obesity-associated coronary heart disease (CHD) continue to face significant residual risk (1, 2). This residual risk is defined as the sustained elevation in the incidence of major adverse cardiovascular events despite adherence to guideline-directed optimal medical therapy (2). Epidemiological evidence underscores that obesity is not merely a co-occurrence of risk factors but acts as an independent and potent pathophysiological amplifier, perpetuating a systemic milieu conducive to both atherogenesis and thrombosis (3, 4).

The current therapeutic paradigm, which primarily targets cholesterol reduction and platelet inhibition, exhibits a discernible “ceiling effect” (1, 5). A key limitation is its predominant focus on mitigating downstream manifestations of disease—such as plaque progression and thrombus formation—while inadequately addressing upstream pathogenic drivers (6). The metabolic dysfunction induced by obesity encompasses a far more complex, system-wide network (7, 8). Central to this network are the intertwined processes of metabolic dysregulation and chronic low-grade inflammation, which are frequently studied in isolation. Traditional research often delineates investigations into metabolic disturbances (e.g., insulin resistance, ectopic lipid deposition) from those examining inflammatory pathways (e.g., mediated by monocytes or inflammasomes) (6–8). This fragmented approach fails to capture their essential bidirectional interaction and synergy.

Consequently, a paradigm shift is warranted: from targeting discrete pathways to implementing integrated strategies that address the underlying pathological network (9, 10). This perspective article posits the “inflammation-metabolism” axis as a unifying framework for understanding residual risk in obesity-related CHD. Within this model, dysfunctional adipose tissue releases excessive fatty acids, adipokines, and other mediators that concurrently disrupt metabolic homeostasis and activate innate immune signaling, thereby establishing a self-perpetuating cycle (9). Conversely, inflammatory pathways can directly impair insulin signaling, further exacerbating metabolic imbalance (11). This vicious cycle continuously fuels atherosclerotic plaque development and instability (11).

To further clarify its conceptual positioning, the inflammation–metabolism axis should be distinguished from related but distinct constructs. Immunometabolism is primarily a foundational research field that examines the interplay between metabolic processes and immune cell function at molecular and cellular levels (12). Metabolic inflammation, or meta-inflammation, refers to a chronic low-grade inflammatory state driven by metabolic disturbances, particularly in obesity (13). In contrast, the inflammation–metabolism axis proposed here is a systems-level translational framework that integrates these established mechanisms into a unified pathological network. It emphasizes the dynamic, bidirectional feedback loop between metabolism and inflammation as a clinically actionable target, rather than as a purely descriptive biological phenomenon. Importantly, this axis should not be interpreted as a newly identified biological mechanism, but rather as a translational conceptual framework that reframes well-established immunometabolic interactions into a clinically actionable model. Its primary contribution lies in shifting the focus from isolated pathways to network-level interactions, thereby providing a systems-oriented rationale for developing multi-target intervention strategies aimed at reducing residual cardiovascular risk (13, 14).

**Figure 1:**
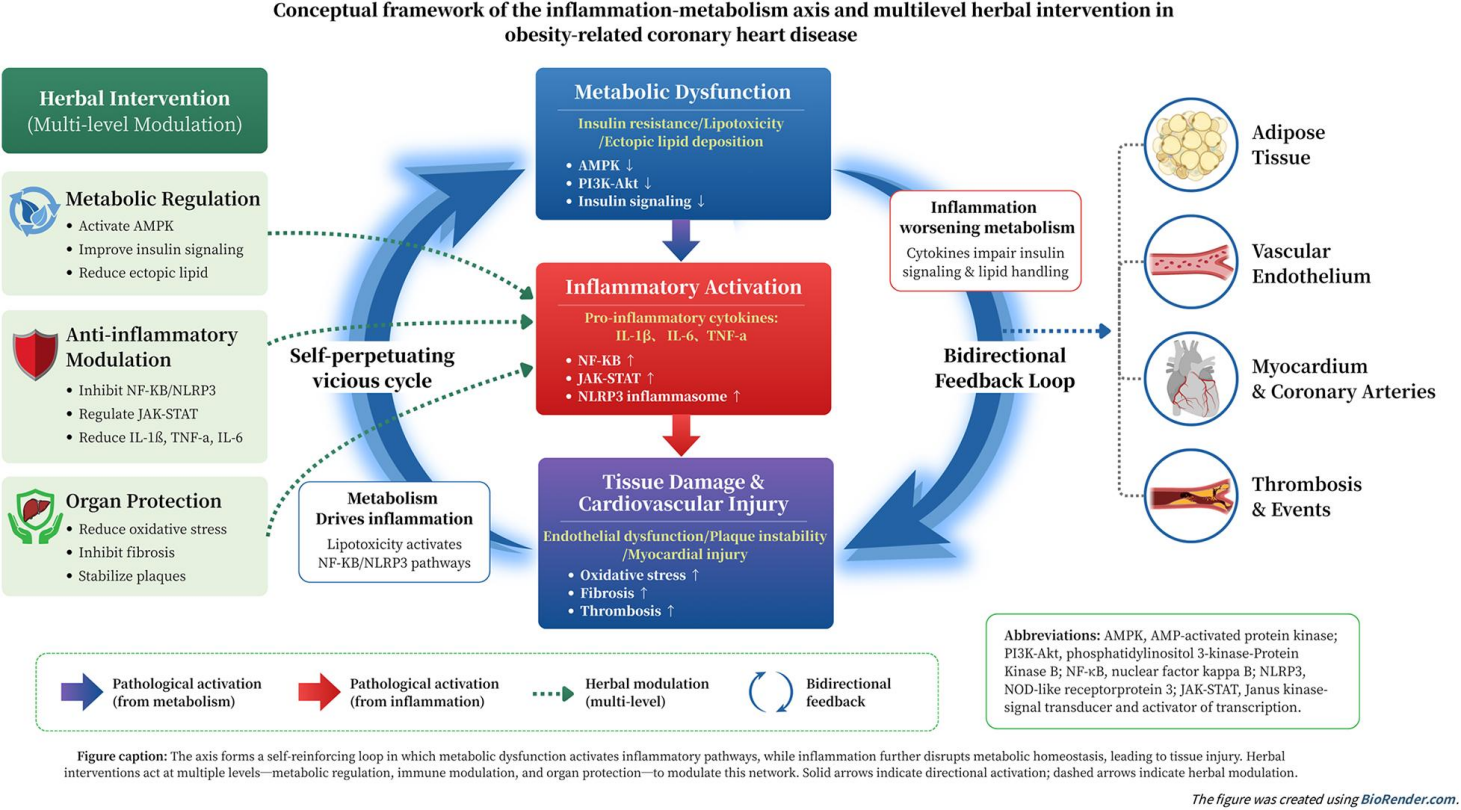
The axis forms a self-reinforcing loop in which metabolic dysfunction activates inflammatory pathways, while inflammation further disrupts metabolic homeostasis, leading to tissue injury. Herbal interventions act at multiple levels-metabolic regulation, immune modulation, and organ protection to modulate this network. Solid arrows indicate directional activation; dashed arrows indicate herbal modulation.

Building upon this integrated pathophysiological perspective, therapeutic interventions capable of simultaneously modulating multiple nodes within the inflammation-metabolism network are of particular interest (15). Herbal drug preparations, characterized by their multi-component and multi-target properties, represent a promising strategy in this context (16). Through coordinated regulation of metabolic and inflammatory pathways, such polypharmacological interventions may disrupt the core pathological cycle and offer a complementary approach for reducing residual cardiovascular risk in this high-risk population (17, 18). The conceptual framework of the inflammation–metabolism axis and its modulation by multi-level herbal interventions is illustrated in Figure 1.

## 2 Inflammation-Metabolism axis: A pathological Hub of residual cardiovascular risk

Building on existing concepts in immunometabolism and metabolic inflammation, the inflammation–metabolism axis extends beyond descriptive categorization by emphasizing network-level organization and therapeutic relevance (19). Rather than focusing solely on molecular interactions or inflammatory states, this framework highlights how these processes are dynamically integrated into a self-reinforcing pathological circuit that drives disease progression and residual cardiovascular risk.

### 2.1 A self-sustaining pathogenic circuit

The inflammation–metabolism axis constitutes an integrated and dynamically coupled pathogenic system. It goes beyond traditional concepts of immunometabolic interactions or metabolic inflammation by explicitly framing their bidirectional feedback as a self-sustaining disease network. In obesity, persistent caloric excess, insulin resistance, and the accumulation of lipotoxic agents (e.g., ceramides, saturated fatty acids) establish a state of metabolic dysregulation (9). These metabolic disturbances directly trigger innate immune activation via pathways including Toll-like receptor 4 signaling and reactive oxygen species generation. A critical convergence point is the NOD-like receptor family pyrin domain containing 3 (NLRP3) inflammasome, whose activation leads to the secretion of potent pro-inflammatory cytokines, primarily interleukin-1β (IL-1β) and interleukin-18 (6). Concurrently, immune cells—notably macrophages—infiltrate adipose tissue and adopt a pro-inflammatory phenotype, amplifying the inflammatory response (20). Crucially, this resulting inflammatory milieu actively worsens metabolic function. Cytokines such as tumor necrosis factor-α, IL-1β, and interleukin-6 impair insulin signaling (e.g., via inhibitory serine phosphorylation of insulin receptor substrate-1), promote lipolysis, and drive ectopic lipid deposition in the liver, muscle, and heart (20). Thus, a vicious cycle is established: metabolic dysfunction fuels inflammation, which in turn exacerbates metabolic dysregulation.

### 2.2 Downstream consequences: From molecular pathways to organ damage

The persistent activity of this axis drives cardiovascular damage at multiple levels. At the vascular level, it induces endothelial dysfunction by reducing nitric oxide bioavailability and upregulating adhesion molecules, thereby accelerating atherosclerosis (21, 22). Within atherosclerotic plaques, the inflammatory-metabolic environment promotes features of vulnerability: it weakens the fibrous cap, inhibits collagen synthesis, and increases matrix metalloproteinase activity (23). Beyond the vasculature, the heart itself becomes a target. Systemic and local inflammatory signals, combined with myocardial insulin resistance and lipotoxicity, contribute to cardiomyocyte apoptosis, interstitial fibrosis, and metabolic disturbances in cardiac energy production (24, 25). This process underlies the development of a specific clinical entity—insulin-resistant cardiomyopathy—which further elevates the risk of heart failure independent of coronary obstruction (24).

### 2.3 Mechanistic basis for unresolved risk

This axis provides the fundamental explanation for persistent residual risk despite optimal conventional therapy. Current cornerstone treatments, such as high-intensity statins or proprotein convertase subtilisin/kexin type 9 inhibitors, are highly effective at lowering low-density lipoprotein cholesterol but only partially address the interconnected inflammatory and metabolic network (26, 27). Similarly, antiplatelet agents target the final step of thrombosis without disrupting the upstream inflammatory drivers of plaque instability (27). The inflammation-metabolism axis functions as a resilient, adaptive network; interventions against a single component are often insufficient to halt the entire circuit (28, 29). Consequently, its ongoing activity offsets some benefits of standard therapies, allowing risk to persist (27, 28). Therefore, effectively reducing residual risk requires a strategic shift from single-target approaches to interventions capable of modulating this core pathological network.

## 3 Herbal medicine as a systemic therapeutic tool targeting the “inflammation-metabolism” axis

### 3.1 Theoretical advantages of multi-component synergistic therapy

Effective intervention against the self-perpetuating inflammation-metabolism axis requires a strategy that moves beyond single-pathway inhibition (30). Herbal medicines, with their intrinsic multi-constituent nature, align with the principles of network pharmacology (31). This framework suggests that the combined, often synergistic action of multiple phytochemicals—each with modest individual effects—can collectively modulate broad pathological networks. This “multi-target, moderate-effect synergy” is particularly suitable for managing complex systemic diseases such as obesity-associated CHD (30). Unlike highly specific single-target drugs, which may be bypassed by network adaptations, a well-defined herbal preparation can simultaneously engage several key nodes within the axis (32). This multi-pronged approach may restore systemic homeostasis more effectively by addressing the multifaceted origins of the disease (32).

### 3.2 Hierarchical actions of herbal medicine in modulating the axis

The therapeutic potential of herbal medicine in modulating the inflammation–metabolism axis can be conceptualized across three complementary and hierarchically organized levels of intervention, reflecting its capacity to act on interconnected biological networks rather than isolated pathways (Table 1). Importantly, the individual phytochemicals discussed below are presented primarily to illustrate specific mechanistic nodes within the inflammation–metabolism axis, rather than to represent the full therapeutic paradigm of herbal medicine. In real-world clinical practice, herbal interventions are typically administered as multi-component formulas or standardized extracts, in which synergistic and network-level interactions among multiple constituents give rise to systemic therapeutic effects (33). Therefore, the actions described at each level should be interpreted as components of an integrated systems-level response rather than independent single-compound effects.

**Table 1:** Multi-component herbal formulations targeting the inflammation–metabolism axis: a systems-level network framework.

Intervention level	Representative herbal formula/extract	Core component clusters	Key network targets	Systems-level functional effects	Translational relevance
Upstream metabolic regulation	Gegen Qinlian Decoction, Berberine-containing Coptis-based formulas	Alkaloids (berberine), flavonoids, glycosides	AMPK activation PI3K–Akt signaling, Mitochondrial metabolism	Restoration of insulin Sensitivity, Reduction of Lipotoxicity, Energy metabolism reprogramming	Targets metabolic root of residual risk
Immune–inflammatory modulation	Astragalus-based formulas, Gegen Qinlian Decoction	Polysaccharides, flavonoids, saponins	NF-*κ*B pathway, NLRP3 Inflammasome, JAK–STAT signaling	Suppression of chronic low-grade inflammation, Macrophage polarization (M1→M2), Cytokine network rebalancing	Targets inflammatory amplification loop
End-organ protection	Danshen-based formulations, Compound Danshen preparations	Phenolic acids (salvianolic acids), diterpenoids	Oxidative stress pathways, Endothelial signaling, Fibrosis-related pathways (TGF-*β*)	Endothelial protection, Plaque stabilization, Anti-fibrotic remodeling	Targets vascular and myocardial injury
Integrated axis modulation	Multi-herb combination formulas (e.g., cardiometabolic TCM prescriptions integrating above modules)	Multi-class phytochemical networks	Cross-talk between Metabolic + inflammatory pathways, Network-level reprogramming	Disruption of inflammation–metabolism feedback loop, Systemic homeostasis restoration	Aligns with residual risk reduction strategy

AMPK, adenosine monophosphate-activated protein kinase; PI3K, phosphoinositide 3-kinase; Akt, protein kinase B; NF-κB, nuclear factor kappa B; NLRP3, NOD-like receptor family pyrin domain containing 3; JAK, Janus kinase; STAT, signal transducer and activator of transcription; TGF-β, transforming growth factor beta; TCM, traditional Chinese medicine.

#### 3.2.1 Level 1: upstream metabolic regulation

At the upstream level, herbal interventions target the core metabolic disturbances that initiate and sustain the pathological cycle. Representative phytochemicals such as berberine (derived from *Coptis chinensis*) and resveratrol have been shown to enhance insulin sensitivity through activation of key metabolic regulators, including adenosine monophosphate-activated protein kinase and phosphoinositide 3-kinase/protein kinase B signaling pathways (34). In parallel, compounds such as curcumin modulate lipid metabolism via nuclear receptors (e.g., peroxisome proliferator-activated receptors) while improving mitochondrial function, thereby addressing fundamental bioenergetic dysfunction associated with metabolic syndrome (35, 36). Collectively, these effects contribute to the restoration of metabolic homeostasis and attenuation of lipotoxic stress.

#### 3.2.2 Level 2: immune-inflammatory modulation

At the intermediate level, herbal agents act on central inflammatory hubs that amplify the pathological loop. Bioactive compounds such as curcumin and epigallocatechin gallate suppress pro-inflammatory signaling cascades, including nuclear factor kappa B, Janus kinase/signal transducer and activator of transcription, and NLRP3 inflammasome activation (37). Additionally, certain herbal constituents- such as astragalus polysaccharides- promote macrophage phenotypic switching from a pro-inflammatory (M1) to a reparative (M2) phenotype (38). Beyond direct immune modulation, emerging evidence suggests that herbal-derived fibers and polyphenols can reshape gut microbiota composition, thereby indirectly influencing systemic inflammation through the gut–immune axis (39). These coordinated actions contribute to rebalancing the inflammatory network rather than merely suppressing isolated cytokine pathways.

#### 3.2.3 Level 3: end-organ protection

At the downstream level, herbal compounds mitigate tissue and organ damage resulting from prolonged axis dysregulation. Salvianolic acids from *Salvia miltiorrhiza* exhibit potent antioxidant properties and play a critical role in preserving endothelial function (40). Meanwhile, compounds such as silibinin and tetramethylpyrazine demonstrate protective effects against myocardial fibrosis and plaque stability, respectively, through modulation of oxidative stress and fibrotic signaling pathways (41, 42). These effects collectively contribute to maintaining vascular integrity and cardiac function, thereby limiting the clinical consequences of chronic inflammation–metabolism imbalance.

#### 3.3 Potential for complementary integration with conventional therapies

The systemic, multi-target nature of herbal medicine suggests significant promise for synergistic integration with standard pharmacotherapy (43). While statins effectively lower low-density lipoprotein cholesterol, specific herbals may concurrently ameliorate the vascular inflammatory milieu, potentially reinforcing plaque stabilization (44). In patients receiving anti-diabetic agents, herbals that improve insulin sensitivity and mitochondrial function could offer complementary metabolic benefits (34). Moreover, certain plant-derived compounds may help mitigate select side effects of conventional drugs; for instance, hepatoprotective herbals could provide adjunctive support during long-term statin therapy (45). Ultimately, a rationally designed integrative approach, combining evidence-based herbal formulations with guideline-directed care, may enable a more comprehensive suppression of the inflammation-metabolism axis, contributing to a meaningful reduction in residual cardiovascular risk. Importantly, as shown in Table 1, the therapeutic effects of herbal medicine arise from coordinated, multi-component interactions across multiple biological levels, rather than from the activity of isolated single compounds (31). This systems-level approach aligns with the network structure of the inflammation–metabolism axis.

Emerging clinical evidence provides preliminary support for this integrative therapeutic concept. Randomized and controlled studies have reported that combination strategies incorporating multi-component herbal formulations- particularly those containing *Salvia miltiorrhiza* and *Pueraria lobata*-can improve lipid profiles, endothelial function, and inflammatory markers in patients with hyperlipidemia or coronary heart disease when administered alongside standard pharmacotherapy (46, 47). In parallel, clinical investigations of representative phytochemicals, such as curcumin, have demonstrated beneficial effects on metabolic parameters, including insulin resistance and lipid metabolism, in individuals with metabolic syndrome (48, 49). These findings offer an initial clinical foundation for the complementary role of herbal medicine within cardiometabolic management.

Nevertheless, the current body of clinical evidence remains limited and should be interpreted with caution. Existing studies are often constrained by relatively small sample sizes, heterogeneity in herbal formulations, and variability in study design, endpoints, and duration (50, 51). In addition, robust data on long-term cardiovascular outcomes are still lacking (52). These limitations underscore the necessity for rigorously designed, large-scale, and biomarker-informed clinical trials to validate both the efficacy and safety of multi-component herbal interventions within the inflammation–metabolism axis framework, thereby strengthening their translational credibility. These findings provide an initial clinical basis for further validation of the inflammation–metabolism axis within biomarker-driven, systems-level therapeutic frameworks.

## 4 From paradigm to practice: research strategies and a translational roadmap

To enhance translational clarity and practical applicability, the development of herbal interventions targeting the inflammation–metabolism axis can be structured into a stepwise and iterative pipeline. This process encompasses: (1) systems biology–driven discovery of candidate multi-component formulations; (2) mechanistic validation through network pharmacology and multi-omics approaches; (3) evaluation in advanced preclinical models that recapitulate metabolic–immune interactions; (4) biomarker-guided early-phase clinical trials to confirm target engagement; and (5) large-scale randomized clinical trials to assess efficacy and safety (53, 54). Such a structured framework establishes a clear and continuous progression from conceptual discovery to clinical implementation, while enabling iterative refinement across stages.

### 4.1 Systems biology–driven discovery and validation

Translating the “inflammation-metabolism” axis concept into clinical application demands systems-level methodologies that can clarify how herbal medicines operate across entire biological networks (16, 55). The central approach should integrate network pharmacology—combining computational modeling with experimental validation—and multi-omics analyses (e.g., transcriptomics, metabolomics, proteomics) to systematically map how herbal formulations concurrently influence key nodes in both metabolic and inflammatory pathways (16, 55). This moves beyond single-target studies by examining interactions between two complex systems: the human pathological network and the multi-component chemical system of the herbal agent (16).

At the initial discovery stage, candidate herbal formulations should be systematically screened using network pharmacology and computational modeling to identify key component clusters and their predicted interactions with metabolic and inflammatory pathways (18, 56). These in silico predictions should then be subjected to stepwise experimental validation using multi-omics approaches, enabling the identification of critical network nodes, pathway-level perturbations, and emergent system-level effects (56, 57). This sequential strategy establishes a structured transition from computational hypothesis generation to experimentally supported mechanistic insight, thereby enhancing both the robustness and reproducibility of early-stage discovery.

From a hypothesis-driven perspective, the inflammation–metabolism axis framework can be operationalized through several testable propositions that guide both experimental design and clinical translation. First, coordinated modulation of both metabolic and inflammatory nodes is expected to achieve greater reduction in residual cardiovascular risk compared with single-target interventions, reflecting the network-based nature of the underlying pathology (58, 59). Second, the activity of the axis—quantified through integrated biomarker panels encompassing metabolic and inflammatory signatures—should correlate with disease severity and clinical outcomes, thereby providing a measurable indicator of therapeutic response (60). Third, multi-component herbal interventions are hypothesized to induce system-wide network reprogramming, which can be captured and validated through multi-omics profiling approaches (61, 62).

To further strengthen the quantitative and translational validity of this framework, the activity of the inflammation–metabolism axis can be operationalized using integrated biomarker panels that jointly capture metabolic and inflammatory dimensions (60). These may include composite signatures derived from metabolomic–proteomic integration or indices reflecting coordinated dysregulation of cytokine and lipid pathways (63). Such measurable parameters enable dynamic monitoring of axis activity over time and provide an objective basis for assessing therapeutic efficacy. In parallel, computational network modeling approaches that integrate multi-omics datasets can simulate system-level perturbations induced by multi-component interventions, thereby offering a quantitative platform for hypothesis testing, mechanistic inference, and predictive validation (61, 64).

To further enhance mechanistic resolution, advanced high-dimensional techniques such as spatial transcriptomics and single-cell sequencing should be incorporated (65, 66). These approaches enable precise mapping of cell-type-specific and microenvironment-dependent responses, particularly within key pathological sites such as atherosclerotic plaques and metabolically active adipose tissue (67). By capturing how herbal interventions reshape the dynamic crosstalk between immune cells (e.g., macrophage subsets) and metabolic cells (e.g., adipocytes and endothelial cells), such strategies offer a powerful means to validate network-level effects *in situ* and to refine mechanistic understanding at a systems scale.

### 4.2 Development of innovative preclinical models

Current animal models often fail to adequately capture the systemic, interactive nature of the human “inflammation-metabolism” axis in obesity-related CHD (68). Advancing translational research therefore requires more sophisticated preclinical models (69). This includes genetically engineered models that combine features of metabolic syndrome with defined immune perturbations (e.g., obese mice with selective immune-cell deficiencies), as well as diet-induced models supplemented with chronic low-grade inflammatory stimuli (70). Such models more accurately replicate the core pathophysiology—wherein metabolic dysfunction and chronic inflammation perpetuate each other—and thus offer a more predictive platform for evaluating how candidate herbal interventions modulate the axis as an integrated system and confer end-organ protection (71).

Within this translational framework, preclinical models should be positioned as a critical intermediate validation step bridging mechanistic discovery and clinical application (72). Specifically, they enable controlled assessment of system-level therapeutic effects, allowing simultaneous evaluation of metabolic regulation, inflammatory modulation, and organ-level outcomes (73). To fulfill this role, model design should explicitly incorporate both metabolic and inflammatory dimensions, thereby ensuring alignment with the core architecture of the inflammation–metabolism axis (74). Such integrative modeling not only facilitates mechanistic validation but also provides functional evidence supporting the efficacy and feasibility of candidate interventions prior to clinical translation.

### 4.3 Design of the clinical translation pathway

Clinical translation should proceed through a stepwise, biomarker-informed strategy. Initial phase II trials should adopt a biomarker-enriched design, with primary endpoints focused not merely on conventional lipid measures but on validated indicators of axis activity (75, 76). These may include adiponectin, IL-1β, the integrated inflammatory marker GlycA, or specific metabolomic/proteomic signatures. This stage is primarily intended to establish target engagement and to characterize pharmacodynamic modulation of the axis (28).

From a translational perspective, clinical evaluation should be explicitly organized into sequential phases with distinct objective. Early-phase trials should prioritize biomarker-based endpoints to verify mechanistic activity and pathway engagement, whereas later-phase trials should shift focus toward hard clinical outcomes, such as major adverse cardiovascular events, thereby establishing direct clinical relevance (77–79). This phased transition ensures that mechanistic validation is systematically linked to outcome-based efficacy assessment.

Building upon early-phase findings, subsequent pivotal trials may incorporate adaptive design strategies to assess herbal interventions as adjuncts to guideline-directed therapies, including intensive lipid-lowering and antiplatelet regimens, in high-risk populations with obesity-related CHD (80). Such designs enable dynamic modification of study parameters based on interim analyses, thereby improving trial efficiency and responsiveness to emerging data. The ultimate objective is to determine whether such integrative treatment meaningfully reduces residual cardiovascular risk, thereby closing the translational loop from mechanistic insight to measurable clinical benefit (81).

Importantly, these clinical strategies should not be viewed in isolation but rather as integral components of a broader, iterative validation framework. Specifically, clinical trial findings should be systematically integrated with upstream mechanistic studies and downstream outcome assessments to establish a closed-loop validation architecture (82). Within this framework, biomarker-defined changes in inflammation–metabolism axis activity are continuously correlated with clinical endpoints, enabling dynamic refinement of both therapeutic strategies and mechanistic understanding (83, 84). This integrative approach transforms the proposed axis from a conceptual construct into a clinically testable, quantitatively assessable, and progressively optimized therapeutic framework.

Together, these sequential and interconnected steps constitute a continuous and iterative translational pathway, in which evidence generated at each stage feeds back to refine both mechanistic hypotheses and clinical strategies. This integrated pipeline not only enhances the internal coherence of the translational process but also ensures that the proposed inflammation-metabolism axis evolves from a conceptual construct into a clinically actionable, systematically validated, and progressively optimized intervention framework.

## 5 Challenges and future directions

### 5.1 Key scientific challenges

Advancing herbal medicine for the “inflammation-metabolism” axis faces three core scientific hurdles. First, its inherent complexity makes it difficult to identify the critical active components and clarify how they interact within multi-constituent extracts (85). Second, while research has focused on isolated compounds and known pathways, a systems-level understanding of how a full herbal preparation holistically reshapes the disease network remains underdeveloped, especially concerning tissue-specific and systemic integration (86). Third, ensuring consistent clinical outcomes depends on overcoming significant standardization and quality control issues, stemming from natural source variability, differing extraction methods, and analytical challenges in quantifying multi-component mixtures (87).

In addition to these mechanistic and standardization challenges, safety considerations represent a critical and often underappreciated barrier to clinical translation (88, 89). The presence of multiple bioactive constituents within herbal formulations may introduce risks of cumulative toxicity and unintended off-target effects, particularly when pharmacodynamic interactions are not fully characterized (90). Furthermore, herb–drug interactions—especially those mediated by cytochrome P450 enzymes or membrane transporters—can alter the pharmacokinetics and therapeutic profiles of co-administered cardiovascular medications, including statins and antiplatelet agents (91, 92). Addressing these risks necessitates not only rigorous standardization but also systematic pharmacokinetic evaluation and the incorporation of herb–drug interaction assessments into clinical trial design (93, 94).

Beyond these established concerns, it is important to recognize that safety challenges are inherently intertwined with the multi-component nature of herbal medicine itself (95). The simultaneous presence of numerous active constituents may lead to complex interaction patterns, including additive or synergistic toxicity, as well as unanticipated off-target biological effects (95, 96). Moreover, compositional variability across different preparations can further amplify these risks, complicating both dose standardization and safety prediction (87). Therefore, future research should adopt an integrated evaluation framework that combines pharmacokinetic profiling, interaction mapping, and longitudinal safety monitoring within clinical studies. Such an approach is essential to balance the therapeutic advantages of multi-target interventions with the requirements of safety, reproducibility, and translational feasibility.

### 5.2 Regulatory and perception barriers

Translational efforts confront unique barriers in regulation and professional perception (97). Regulatory frameworks, primarily designed for single-molecule drugs, rely on evidence like single-target mechanisms and linear pharmacokinetics (98). A major obstacle is translating evidence of multi-target, systemic effects—such as network pharmacology or multi-omics data—into a language of “efficacy” and “safety” that fits established regulatory paradigms (99). This necessitates a parallel shift in perception: redefining herbals from vaguely defined supplements toward standardized, multi-component therapeutic agents with progressively clarified mechanisms of action, a view that must gain acceptance among scientists, clinicians, and regulators alike (100).

### 5.3 Future directions

To address these challenges, the field must pursue several integrated paths. Future discovery research should move beyond studying single compounds. Leveraging systems biology and computational approaches, researchers can analyze effective traditional formulas to identify novel, rationally designed multi-molecular combinations with optimized synergy (16, 101). In clinical translation, developing biomarker-driven patient stratification is crucial. Utilizing specific axis biomarkers (e.g., metabolomic or inflammatory proteomic signatures) will enable the selection of enriched patient populations, thereby increasing trial precision and therapeutic relevance (102). Finally, overcoming these complex, interdisciplinary challenges requires fostering robust international collaboration to establish consensus on research standards, evaluation methods, and data sharing, accelerating the translation of this integrative paradigm into clinical practice (103).

## 6 Summary

The “inflammation-metabolism” axis offers an integrative and mechanistic framework essential for understanding and addressing residual risk in obesity-related coronary artery disease. This model elucidates a self-reinforcing pathological cycle wherein chronic inflammation and metabolic dysfunction interact to sustain atherosclerotic progression and plaque instability—a core mechanism underlying the limitations of current single-target therapies. Consequently, targeting this axis represents a necessary paradigm shift from conventional reductionist approaches toward strategies capable of modulating systemic disease networks.

In this context, herbal medicines with inherent multi-target properties hold dual significance: they are not only promising therapeutic agents for system-level intervention but also serve as valuable scientific tools for validating this new paradigm and advancing network pharmacology principles. Through rigorous modern research—employing systems biology, advanced preclinical models, and biomarker-enriched clinical trials—the study of herbals may catalyze the development of novel therapies for residual cardiovascular risk and help usher in a more holistic era of systems-based cardiovascular medicine.

## Data Availability

The original contributions presented in the study are included in the article/Supplementary Material, further inquiries can be directed to the corresponding author/s.
